# Effects of Anticoagulants on Experimental Models of Established Chronic Liver Diseases: A Systematic Review and Meta-Analysis

**DOI:** 10.1155/2020/8887574

**Published:** 2020-12-11

**Authors:** Rui Zhang, Xiaoquan Huang, Yingyi Jiang, Jian Wang, Shiyao Chen

**Affiliations:** ^1^Department of Gastroenterology and Hepatology, Zhongshan Hospital, Fudan University, Shanghai, China; ^2^Center of Evidence-based Medicine, Fudan University, Shanghai, China; ^3^Shanghai Institute of Liver Disease, Shanghai, China; ^4^Endoscopy Center and Endoscopy Research Institute, Zhongshan Hospital, Fudan University, Shanghai, China

## Abstract

**Objective:**

The role of anticoagulants in chronic liver diseases is inconclusive. A meta-analysis was thus undertaken to evaluate treatment-related survival and antifibrotic effects in animal models of chronic liver diseases.

**Methods:**

A systematic search of the literature took place (up to November 2020), screening for preclinical studies that evaluated anticoagulant effects in animal models of chronic liver diseases. We assessed the quality of methods and the certainty of evidence. Data on outcomes were extracted and pooled into random-effects models.

**Results:**

Sixteen studies proved eligible, each assessing anticoagulant use in animals with chronic liver diseases. Generally, the pooled evidence demonstrated that the administration of anticoagulants is preventive against fibrogenesis, as indicated by METAVIR fibrosis scores (risk ratio = 0.66, 95% confidence interval: 0.47 to 0.94), portal pressure determinations (mean difference = −1.39, 95% confidence interval: −2.33 to −0.44), inflammatory activity (mean difference = −169.69, 95% confidence interval: −257.64 to −81.74), and indices of hepatic injury, specifically alanine aminotransferase (mean difference = −82.7, 95% confidence interval: −107.36 to −58.04), aspartate aminotransferase (mean difference = −186.12, 95% confidence interval: −254.90 to −117.33), albumin (mean difference = 0.59, 95% confidence interval: 0.16 to 1.01), and total bilirubin (mean difference = −0.96, 95% confidence interval: −1.46 to −0.46), and it had no impact on animal survival (risk ratio = 1.03, 95% confidence interval: 0.94 to 1.13).

**Conclusions:**

Our assessments indicate that in animal models of chronic liver diseases, anticoagulants may alleviate the degree of fibrosis evaluated by the METAVIR score system, portal pressure, inflammatory activity, and serum indices of hepatocellular injury, without impacting survival. High-quality experimental studies are still required.

## 1. Introduction

The liver plays a crucial role in the global hemostatic process through its synthesis and regulation of most pro- and anticoagulant (AC) factors [1]. However, hemostasis may be profoundly disrupted as pathological changes develop in the liver. Fibrosis or cirrhosis shares the final response of the liver to a variety of offending stimuli, as well as a key driver of the natural course of all types of advanced liver disease. Although antifibrotic treatment is a research and a clinical priority, there are presently no drugs licensed antifibrotic drugs for use in humans. Patients with either cirrhosis or NAFLD often are at increased risk of prothrombotic conditions [2, 3], as exemplified by the presence of thrombotic occlusion of the portal vein and microthrombotic occlusion of intrahepatic veins and sinusoids in cirrhotic livers, and thrombotic events including portal vein thrombosis (PVT) in NAFLD and its evolutive forms [4, 5]. In addition, thrombosis of intrahepatic vessels is frequently followed by hepatic inflammatory injury, which may eventually aggravate the progression of fibrosis, and leading to worsening of PVT and portal hypertension (PP) [6, 7]. Mechanistically, the activated hepatic stellate cells (HSCs) are the key pathogenic initiators of hepatic fibrogenesis. They can be activated by thrombin and FXa and inhibited by anticoagulants to prevent or reduce fibrogenesis [5]. These notions support the potential association between coagulation and fibrosis, and the rationale for the use of anticoagulants that rebalance coagulative parameters as an antifibrotic agent and, ultimately, to prevent PVT and PP.

Anticoagulant therapies, such as heparin (standard or low molecular weight formulations), vitamin K antagonists, antiplatelet agents, and novel direct oral ACs (NOACs), are widely available for clinical use to interpret the therapeutic effects of ACs in the context of chronic liver diseases. However, the results also remain inconclusive. Chung et al. reported that warfarin administration significantly enhanced therapeutic response rates in patients with cirrhosis and nontumor-related portal vein thrombosis (PVT), compared with controls [8]. Similarly, one RCT also showed that prophylactic administration of enoxaparin for a year improved survival, and prevented PVT development and hepatic decompensation in patients with advanced compensated cirrhosis [9]. On the contrary, Chen et al. failed to demonstrate any benefit to warfarin use in patients with liver cirrhosis [10]. A previously conducted systematic review and meta-analysis of clinical trials addressed the safety and efficacy of AC treatment in patients with cirrhosis and PVT, but the analysis mainly focused on thrombotic recanalization and hepatic complications [11].

In experimental animal models, anticoagulant therapies have also recently emerged as attractive choices to manage a variety of hepatic injuries inflicted by western diets, bile duct ligation (BDL), and carbon tetrachloride (CCl_4_) or thioacetamide (TAA) toxicity [12, 13]. In addition to their antithrombotic properties, ACs confer antifibrotic and anti-inflammatory effects [14, 15]. The underlying cellular and molecular mechanisms appear to involve activities of hepatic stellate cells (HSCs), endothelial dysfunction, the factor Xa or thrombin, transforming growth factor-beta (TGF-*β*)/SMAD signaling, extracellular signal-regulated kinase (ERK) signaling, signal transduction from AKT to c-RAF, AKT signaling, and the nitric oxide (NO) pathway [13, 16–20]. To date, animal models addressing the effects of ACs on chronic liver diseases have also produced inconclusive results. Cerini et al. showed that enoxaparin reduced liver fibrosis and PP in rats with cirrhosis [21]. Conversely, in models of rats with advanced cirrhosis, enoxaparin did not ameliorate liver function, liver fibrosis, or PP [22]. More importantly, no data on animal models involving chronic liver injuries and anticoagulation have been systematically summarized and reported to date.

To clarify this issue, we comprehensively merged existing preclinical evidence to ascertain the effects of anticoagulant therapies on survival, hepatic fibrosis, PP, hepatic injury, and inflammatory response in animals with established and variably induced chronic liver disease.

## 2. Methods

### 2.1. Literature Search and Study Selection

This study adhered to the Preferred Reporting Items for Systematic Reviews and Meta-Analyses (PRISMA) guidelines (Table S1) [23]. Trials selected for review were retrieved through comprehensive searches of specific electronic databases (PubMed, Cochrane Library, and Web of Science) and were limited to articles published between January 2000 and November 2020. The following filters were applied: (rats OR mice OR animal OR experiment OR preclinical) AND (“liver cirrhosis” OR “liver fibrosis” OR “hepatic cirrhosis” OR “liver fibrosis” OR cirrho^*∗*^) AND (anticoagulant OR aspirin OR enoxaparin OR rivaroxaban). Preclinical, controlled comparative studies of animal disease models with chronic liver disease culminating in hepatic fibrosis/cirrhosis and involving anticoagulant therapy (versus no intervention) were targeted. Grounds for exclusion were as follows: (1) nonanimal or nonoriginal studies, (2) noninterventional studies, (3) experimental animals with chronic liver disease and concurrent hepatocellular carcinoma or bowel inflammation, and (4) models of complications secondary to cirrhosis, without underlying chronic liver disease. Two independent reviewers screened eligible publications, first by title and abstract and then by full text. Disagreements were resolved through discussion with or arbitration by a third reviewer until reaching a consensus.

### 2.2. Data Extraction

For each study, data on trial execution (first author, year of publication, country of origin, and study design), baseline animal characteristics (animal number, age, species/strain, weight, and sex), disease model (modeling method), type of regimen (anticoagulation or control, dose, and route), and outcome assessments (primary outcomes: survival, degree of fibrosis, PP; secondary outcomes: parameters of hepatic damage and inflammatory response) were collected by predesigned electronic form. METAVIR fibrosis scores [24] served to gauge hepatic fibrosis, restricting animals to stages F2–F4 for this meta-analysis. Collagen deposition was assessed by Sirius red stain. Numbers extracted or recalculated for meta-analysis included mean, standard deviation (SD), standard error of the mean (SEM), and median values, as well as interquartile range. Descriptive analysis was undertaken if data were not extracted.

### 2.3. Methodological Quality and Risk of Bias Assessments

Two independent reviewers evaluated the quality of data generated by interventional animal studies using the SYRCLE Risk of Bias tool (Systematic Review Center for Laboratory Animal Experimentation, Nijmegen, The Netherlands) [25]. Discrepancies were rechecked by a third person. The checklists entailed the following points: (1) allocation of sequences (adequately generated and applied?), (2) status of study groups (similar at baseline or adjusted for confounders in the analysis?), (3) allocation process (adequately concealed?), (4) animal housing (random throughout the experiment?), (5) caregivers and/or investigators (blinded to each animal intervention during the experiment?), (6) outcome assessments (animals selected at random?), (7) assessor of outcomes (blinded?), (8) incomplete outcome data (adequately addressed?), and (9) study reports (free of selective outcome reporting?). Because many items were reported as “unclear,” we included four other sources of bias pertaining to study randomization at outcome assessment (item 10), randomization at allocation level (item 11), state funding (item 12), and conflicts of interest (item 13). In items 1–9, “Yes” responses indicated a low risk of bias, whereas “No” indicated high risk, and “Unclear” indicated uncertain risk. In items 10–12, “Yes” indicated reported, and “No or unclear” indicated unreported. For item 13, “Yes” indicated a stated conflict of interest, “No” indicated denial, and “Unclear” indicated no mention.

### 2.4. Certainty of Evidence Assessment

The Grading of Recommendations Assessment, Development, and Evaluation (GRADE) method was used to evaluate the quality of evidence for each outcome [26]. Four key components were considered, including methodological limitations, relevance, coherence, and adequacy. Regarding the quality of evidence, we firstly presumed that studies were randomized by design and then pursued degrading evaluations. Grades of evidence were designated as very low quality, low quality, moderate quality, or high quality.

### 2.5. Statistical Analyses

For outcomes other than survival and fibrosis, data were reported as the mean ± SD, converting any data expressed otherwise (i.e., SEM or median and interquartile range) accordingly [27]. Continuous and dichotomous variables were pooled into a generic inverse-variance and random-effects model using Review Manager 5.3 software (Cochrane Collaboration, Copenhagen, Denmark). Statistical heterogeneity (*I*^2^) values assessed across all studies investigated were ranked as follows: <25%, very low; 25–50%, low; 50–75%, moderate; and >75%, high. Subgroup analyses of primary outcome measures were carried out to identify sources of heterogeneity. Statistical significance was set at *p* ≤ 0.05.

## 3. Results

### 3.1. Literature Selection and Study Characteristics

**Figure 1:**
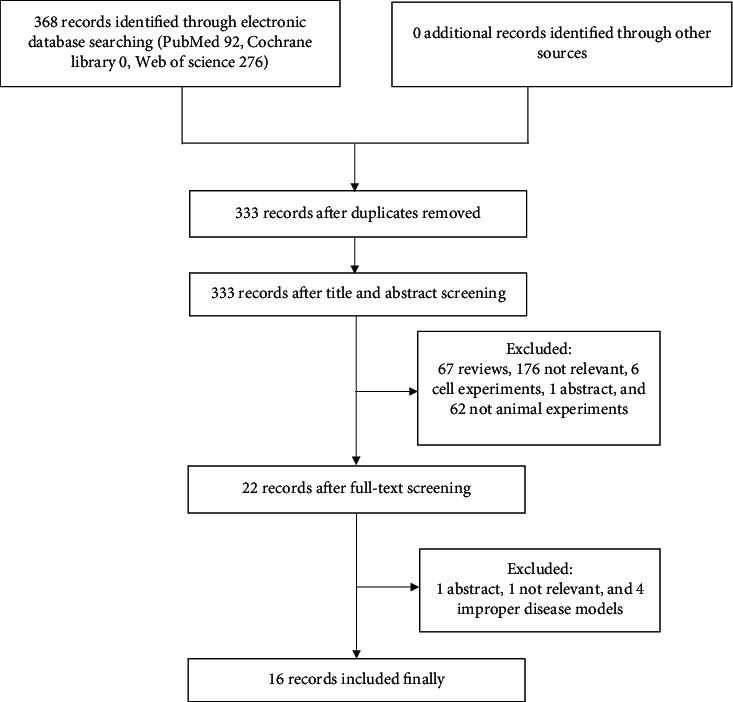
Flow diagram of study selection, searching PubMed, Cochrane Library, and Web of Science (up to November 2020) for studies assessing anticoagulant administration in animal models of chronic liver diseases (total of 16 qualifying publications).

Once deduplication and preliminary screening were complete, a total of 16 publications met our eligibility criteria (Figure 1). Study characteristics are summarized in Table 1 and Table S2. Twelve of these 16 studies were candidates for meta-analysis. The other four presented nonextractable data reserved for descriptive analysis. Cirrhosis, hepatic fibrosis, NAFLD, and cholestatic liver injury were modeled in four, nine, two, and one of the 16 studies, respectively. The most common agents used to induce hepatic fibrosis/cirrhosis were CCl_4_ (75%) and TAA (31.25%). Other modeling strategies included BDL; western diet; choline-deficient, L-amino acid-defined (CDAA) diet; high-fat, high-calorie (HF/HC) diet; porcine serum; and 1% dimethylnitrosamine (DMN). Overall, 75% of animals were male, and 56.25% were Sprague-Dawley rats. Five studies described the effects of low molecular weight heparin (LMWH, enoxaparin) use. Antiplatelet agents (aspirin and clopidogrel) and NOACs (argatroban and rivaroxaban) were applied in five and three studies, respectively. More than one-half of the studies (68.75%) reported joint administration of ACs during animal modeling.

### 3.2. Study Quality and Risk of Bias Assessments

**Figure 2:**
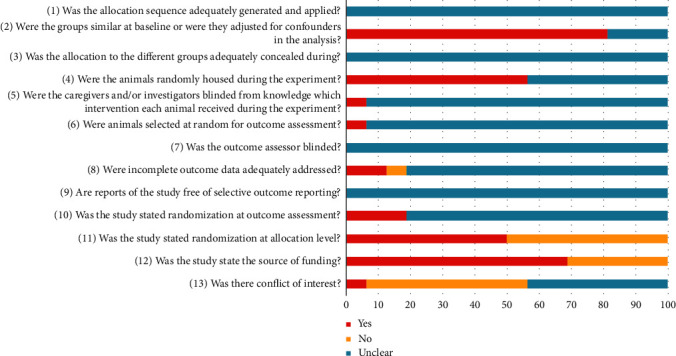
Risk of bias assessments for individual animal studies depicted in a bar chart showing the percentage of all studies that met each quality item, scored as “Yes,” “No,” or “Unclear.”

The risk of bias was evaluated in all 16 studies (Figure 2 and Table S3). Due to poor reporting, most items were viewed as unclear. In assessing selection bias (items 1–3), authors of eight publications merely mentioned randomization, without detailing their procedures; and no authors described allocation concealment. As for performance bias assessment (items 4 and 5), animals were randomly assigned in nine studies, a blinded process cited in one report only. None of the studies mentioned measures for detection bias (item 6 and 7), and most (81.25%) were unclear in terms of attrition bias (item 8). None of the studies outlined a study protocol, so the risk of reporting bias (item 9) was unclear. As shown in Figure 2, three and eight authors referred to randomization at points of outcome assessment (item 10) and allocation (item 11), respectively. Finally, five of these 16 publications (31.25%) did not specify sources of funding (item 12), and conflict of interest declarations (item 13) were largely unclear (43.75%), as opposed to denials (50%) or acknowledgments (6.25%).

### 3.3. Animal Survival and Effect Estimates

**Figure 3:**
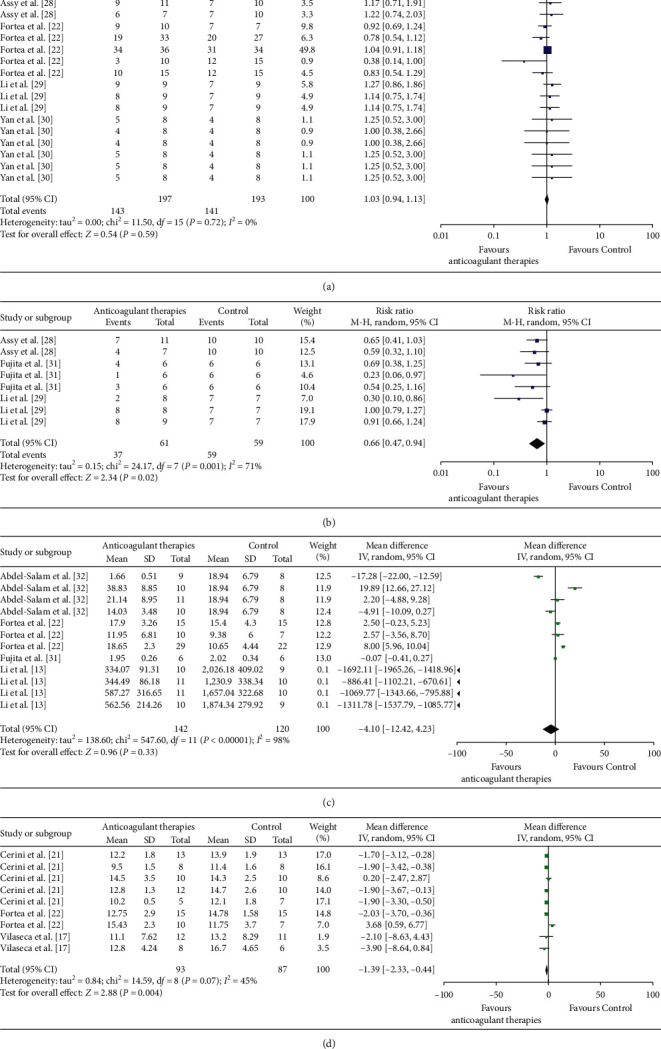
Forest plots in animal models of chronic liver diseases, comparing (a) animal survival, (b) METAVIR fibrosis scores, (c) collagen deposition, and (d) portal pressures after anticoagulant administration.

A total of 16 comparisons involving 390 animals were pooled to investigate animal survival after use of ACs, including low molecular weight heparins (LMWHs) and antiplatelet agent (aspirin) [22, 28–30]. As shown in Figure 3(a), administration of ACs conferred no change in animal survival (risk ratio [RR] = 1.03, 95% confidence interval (CI): 0.94∼1.13), with moderate certainty; and heterogeneity was very low (*I*^2^ = 0%) across all studies. Using subgroup analyses, LMWHs (RR 0.96, 95% CI 0.84∼1.09) and aspirin (RR 1.17, 95% CI 0.90∼1.51) also did not impact animal survival and heterogeneity was very low (*I*^2^ = 0%) across all studies.

### 3.4. Measures of Hepatic Fibrosis and Effect Estimates

Fibrosis evaluations included semiquantitative METAVIR scores and visual gauging of collagen (type I and III) depositions. To assess hepatic fibrosis, eight comparisons involving 120 animals were pooled. Outcomes suggested amelioration in degrees of fibrosis by AC therapies (enoxaparin and aspirin) (RR 0.66, 95% CI 0.47∼0.94), with very low certainty; and heterogeneity was moderate (*I*^2^ = 71%) [28, 29, 31] (Figure 3(b) and Table 2). Subgroup analyses were conducted to examine potential factors contributing to the heterogeneity of total events. All subgroups (see Table 3), including various animal models, species, and treatment parameters (duration, timing, and types of AC), were sources of heterogeneity, for example, the effect sizes of male species (RR = 0.65, 95% CI: 0.4∼1.04; *I*^2^ = 79%), and antiplatelet agent subgroup (RR = 0.58, 95% CI 0.37∼0.91; *I*^2^ = 61%). We further addressed the effect of each anticoagulation separately and found that aspirin has a protective effect on degrees of fibrosis (RR = 0.58, 95% CI 0.37∼0.91; *I*^2^ = 61%) that is not seen with enoxaparin (RR = 0.83, 95% CI 0.50∼1.40; *I*^2^ = 76%).

There were eight comparisons involving 262 animals to assess the effects of ACs on collagen deposition. ACs failed to reduce areas of collagen deposition (mean difference (MD) = −4.10, 95% CI: −12.42∼4.23), with very low certainty [13, 22, 31, 32]; and heterogeneity was high (*I*^2^ = 98%) (Figure 3(c) and Table 2). Among all types of ACs, standard heparin, LMWHs, and antiplatelet agents were involved in evaluating the effects of ACs on collagen deposition; separate outcome suggested that only standard heparin (MD = −986.86, 95% CI −1758.75∼−214.98, *I*^2^ = 99%) reduced areas of collagen deposition, whereas LMWHs (MD = 1.63, 95% CI −6.23∼−9.49, *I*^2^ = 96%) and antiplatelet agents (MD = −0.07, 95% CI −0.41∼0.27) did not.

Nine publications [12, 17, 21, 30, 33–37] stated that chronicled hepatic fibrosis observed significant attenuation in expression levels of alpha-1 type 1 collagen (COL1A1), TGF-*β*1, tissue inhibitor of metalloproteinase-1 (TIMP-1), matrix metalloproteinase-2 (MMP-2), and *α*-smooth muscle actin (*α*-SMA) owing to ACs, such as LMWH (e.g., enoxaparin), NOACs (argatroban and rivaroxaban), thrombin inhibitor (dabigatran), and antiplatelet agent (aspirin). In general, administration of ACs alleviated degrees of hepatic fibrosis in these animal models, proving particularly effective for lowering METAVIR scores and other makers of fibrosis.

### 3.5. Portal Pressure and Effect Estimates

Eight comparisons involving 180 animals addressed the effects of ACs on PP, showing that ACs reduced PP (MD = −1.39, 95% CI −2.33∼−0.44), with low certainty; and heterogeneity was low (*I*^2^ = 45%) [17, 21, 22] (Figure 3(d) and Table 2). All subgroups similarly introduced heterogeneity (Table 4), with one example being the effect size of male species (MD = −1.25, 95% CI −2.27∼−0.23; *I*^2^ = 56%) and LMWH (MD = −1.25, 95% CI −2.27∼−0.23; *I*^2^ = 56%). In all types of ACs, LMWH (enoxaparin) and NOAC (rivaroxaban) were involved in evaluating the association between ACs and PP, and the separate outcome showed that enoxaparin (MD = −1.25, 95% CI −2.27∼−0.23, *I*^2^ = 56%) but not rivaroxaban (MD = −3.28, 95% CI −7.11∼0.56, *I*^2^ = 0%) reduced PP.

### 3.6. Inflammation (Serum TNF-*α*) and Effect Estimates

**Figure 4:**
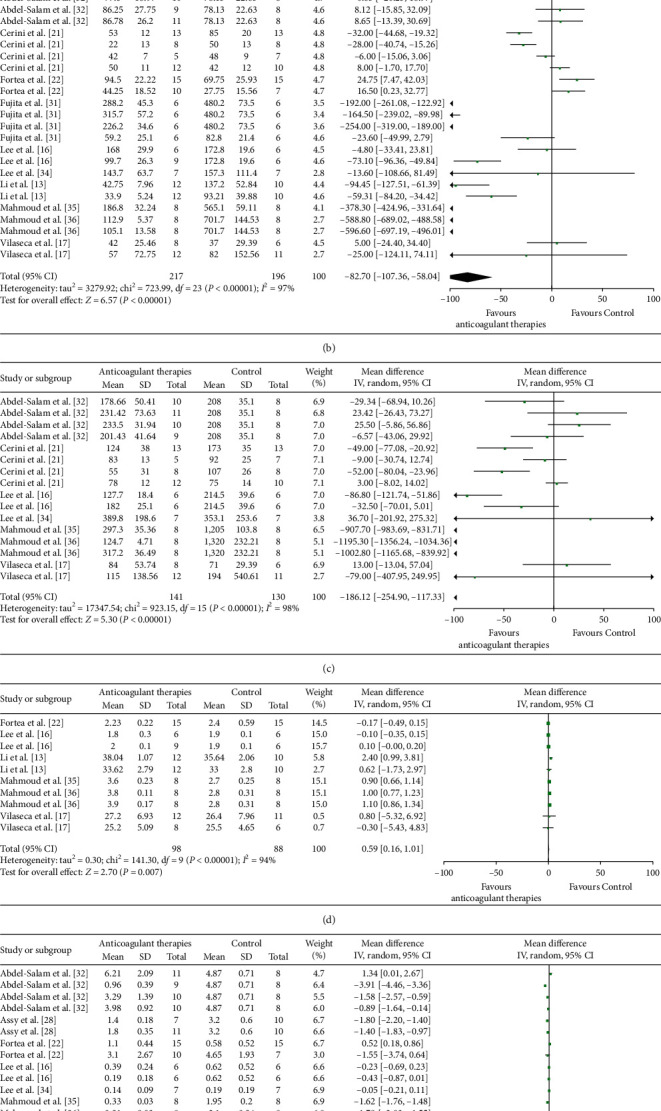
Forest plots in animal models of chronic liver diseases comparing effects of anticoagulant use on serum levels of (a) TNF-*α*, reflecting inflammation, and (b–e) indices of functional hepatic damage (ALT, AST, albumin, and total bilirubin).

Four comparisons involving 48 animals were analyzed to assess the effects of ACs (antiplatelet agents) on serum levels of tumor necrosis factor-alpha (TNF-*α*), which was lower in antiplatelet agents-treated (versus untreated) animals (MD = −169.69, 95% CI: −257.64∼−81.74), with very low certainty [31]; and heterogeneity was high (*I*^2^ = 80%) (Figure 4(a) and Table 2).

Nine studies reported on hepatic inflammatory responses to AC treatment, five of them showing that ACs (argatroban and LMWH) significantly dampened inflammation in the liver [12, 30, 35–37]. Hepatic macrophage and neutrophil accumulation/clustering were diminished as a result of reduced protein expression (CD68, MCP-1, F4/80, ICAM-1, and MIP-2) and cytokine secretion (TNF-*α*, interleukin- (IL-) 6, and IL-1*β*). However, the other four studies failed to credit LMWH (dalteparin and enoxaparin) and thrombin inhibitor (dabigatran) with anti-inflammatory effects [21, 22, 33, 34].

### 3.7. Indices of Hepatic Damage (ALT, AST, Albumin, and Total Bilirubin) and Effect Estimates

Overall, 24 comparisons (in 413 animals) [13, 16, 17, 21, 22, 31, 32, 34–36], 16 comparisons (in 271 animals) [16, 17, 21, 32, 34–36], 10 comparisons (in 186 animals) [13, 16, 17, 22, 35, 36], and 16 comparisons (in 280 animals) [16, 17, 22, 28, 32, 34–36] were undertaken to investigate the effects of AC fluctuations in alanine aminotransferase (ALT), aspartate aminotransferase (AST), albumin, and total bilirubin, respectively, as markers of hepatic injury. Pooled analyses indicated that in AC-treated (versus untreated) animals, liver damage was reduced (ALT: MD = −82.70, 95% CI: −107.36∼−58.04; AST: MD = −186.12, 95% CI: −254.90∼−117.33; albumin: MD = 0.59, 95% CI: 0.16–1.01; total bilirubin: MD = −0.96, 95% CI: −1.46∼−0.46), with very low certainty. However, heterogeneity was high (*I*^2^: ALT, 97%; AST, 98%; albumin 94%; total bilirubin, 98%) (Figures 4(b)–4(e) and Table 2). As shown in Table 5, we further assessed the effect on indices of hepatic damage of each anticoagulation separately. The data supported that only antiplatelet agents improved all serum indices of hepatic function, including ALT, AST, albumin, and total bilirubin.

### 3.8. Sensitivity Analysis

Sensitivity analysis was performed to evaluate the robustness of effect sizes for our pooled outcome estimates by excluding single studies sequentially. For example, the findings of Fortea et al. [22] implied that enoxaparin had no impact on collagen-stained areas. By removing this publication, the pooled effect was altered, showing significantly less collagen deposition due to AC therapy (MD = −31.01, 95% CI: −50.79 to −11.22), so we must be cautious when interpreting this outcome.

## 4. Discussion

In patients with various chronic liver diseases, coagulopathy-related complications often arise in conjunction with hepatic fibrosis and cirrhosis. Intrahepatic thrombosis and hepatic fibrosis are closely associated conditions [38, 39]. Although ACs are widely used to treat or reduce PVT in patients with cirrhosis [40], the effects of antithrombotic therapies on hepatic fibrosis have yet to be fully examined. Current treatments aimed at fibrotic/cirrhotic states and the concomitant complications remain unsatisfactory. Our meta-analysis of preclinical data provides evidence that AC treatment of animals with chronic liver diseases lowers PP, lessens the progression of fibrosis, reduces hepatic inflammation, and improves hepatic function, compared with vehicle-treated controls, which may thus represent a promising tool in chronic liver diseases. To our knowledge, the present meta-analysis is the first to address AC safety and efficacy in animal models simulating the fibrosis/cirrhosis imposed by various chronic liver diseases.

Hepatitis C virus (HCV) and hepatitis B virus (HBV), alcohol abuse, and NAFLD were the leading causes of chronic liver diseases worldwide in clinical cases. Ethanol intake appears as an independent predictor of death in patients with chronic viral liver diseases [41]. In contrast, in our experimental analysis, most (81.25%) models of chronic liver disease were hepatic fibrosis/cirrhosis and were largely induced by CCl_4_ (75%) and TAA (31.25%). There was only one model of cholestatic liver injury as well as two models of NAFLD. In cirrhosis, PVT is a frequent complication, particularly in subjects with moderate–severe liver failure, with a prevalence of 17% [42]. And similar to cirrhosis, NAFLD was also described as the prototypic prothrombotic liver disease due to derangements across all stages of hemostasis, leading to an increased risk of thrombotic events including portal venous and the systemic circulation [4]. In addition, NAFLD may progress to nonalcoholic steatohepatitis- (NASH-) related cirrhosis and appears at higher mortality. Currently, anticoagulant therapy of cirrhosis across the NAFLD spectrum is as yet incompletely defined [5]. Therefore, the experimental model of NAFLD should also ideally be more used in the future to evaluate the impact and safety of anticoagulants.

Either standard or low molecular weight heparin, vitamin K antagonists, antiplatelet agents, and NOACs are a heterogeneous class of drugs. Here, we addressed the effect on all outcomes of each anticoagulant drug separately. Based upon qualifying preclinical trials, we have determined that all types of AC treatments do not impact animal survival, although antiplatelet agents do lower METAVIR fibrosis scores and standard heparin does reduce areas of collagen deposition. HSCs are one kind of liver-resident populations and generally activated in the context of cirrhosis to secrete an excess of extracellular matrix and intensify hepatic vascular tone, contributing to fibrosis progression and portal hypertension [43]. We have observed significant attenuation in expression levels of activated HSCs markers (e.g., COL1A1, TGF-*β*1, TIMP-1, MMP-2, and *α*-SMA) owing to LMWH (e.g., enoxaparin), NOACs (argatroban and rivaroxaban), thrombin inhibitor (dabigatran), and antiplatelet agent (aspirin). In addition, we found lower PP values in enoxaparin-treated animals than in vehicle-treated controls. It is well known that the fibrotic liver is an inflammatory microenvironment which is characterized by infiltrating various leukocytes (e.g., macrophages and neutrophils). In turn, inflammatory injury drives fibrogenesis [43]. According to our results, we found that hepatic macrophage and neutrophil accumulation/clustering were diminished by argatroban and LMWH as a result of reduced protein expression (CD68, MCP-1, F4/80, ICAM-1, and MIP-2) and cytokine secretion (TNF-*α*, IL-6, and IL-1*β*). Likewise, we noted a dampening of inflammation (a regular accompaniment of hepatic fibrosis) [44] by antiplatelet drugs, reflected in diminished expression of TNF-*α*, and normalized levels of various markers (ALT, AST, albumin, and total bilirubin) signaling preservation of hepatic function. Our outcomes were slightly different from clinical studies in which anticoagulants were mainly applied to evaluate survival, the incidence of bleeding events reporting, partial or complete portal venous system recanalization, and the rate of PVT recurrence in patients with cirrhosis [9, 45].

Methodological weaknesses of preclinical studies often result in exaggerated effect sizes [46–48], so we invoked the SYRCLE's risk of bias tool to gauge the quality of these particular efforts. Unfortunately, most checklist items were deemed “unclear” due to inadequate reporting, indicating the poor reporting quality of studies and overestimation of effect sizes. This exercise underscored the need to render adequate quality reporting when conveying preclinical scientific accounts. Moreover, our estimates of effects were tenuous and were rated as very low, low, or moderate certainty in most instances. It is therefore quite likely that subsequent research of this nature will deviate. Furthermore, the heterogeneity of factors in animal models, species, and AC administration (i.e., duration, timing, and type of AC) limited the generalizability of our evidence. For example, the findings of Fortea et al. [22] indicated that enoxaparin does not ameliorate liver fibrosis in rats, and three separate sources [21, 22, 33] reported similar outcomes for systemic inflammation in both LMWH-treated (enoxaparin and dalteparin) and control groups. The results of the three trials differed from those of other studies selected.

Our review has several acknowledged limitations, one being the range of experimental models and different types of injuries represented. Model-dependent mechanisms may certainly impact the conclusions reached. The varied activities and safety profiles of assorted ACs administered were also problematic. A more in-depth investigation into the duration and timing of AC therapeutics is warranted as a consequence. Likewise, the heterogeneity, uncertainty, and small sample sizes confronted in this meta-analysis undermined the validity of the conclusions gathered. Despite our assertion of safety in dispensing ACs for cirrhosis, its conveyance into clinical practice would constitute a sizeable leap. Animals do not share the compensated/decompensated phenotypes of humans.

Based on available animal studies, our findings illustrate the potential benefits of anticoagulant therapy in preventing hepatic fibrosis, portal pressure, inflammatory activity, and serum indices of hepatocellular injury, without impacting survival. More high-quality preclinical studies are still needed in this field.

## Figures and Tables

**Table 1:** Characteristics of included studies.

Study	Study design	Animals	Animal number	Modeling method	Treatment groups	Main outcomes	Courses
Kassel et al. [12]	Unclear	C57BL/6	Unclear	Western diet (40% kcal from milk fat), NAFLD	Vehicle pumps and argatroban pumps	Antifibrotic effect	23 weeks
Li et al. [13]	Unclear	Sprague-Dawley rats	52	CCl_4_/porcine serum, hepatic fibrosis	Normal control, CCl_4_, porcine serum, CCl4 + LAAH, and porcine serum + LAAH	Liver function tests, and area of collagens	10 weeks
Lee et al. [16]	Unclear	Sprague-Dawley rats	24	1% DMN, hepatic fibrosis	Control, DMN, DMN + LH, and DMN + LHP	Antifibrotic effect	4 weeks
Vilaseca et al. [17]	Randomized controlled study	Wistar rats, Sprague-Dawley rats	Unclear	CCl_4_/TAA, cirrhosis	Rivaroxaban and vehicle	Antifibrotic effects, HSC activation, and portal pressure	18 weeks
Cerini et al. [21]	Randomized controlled study	Wistar rats and Sprague-Dawley rats	Unclear	CCl_4_/TAA, cirrhosis	Enoxaparin and vehicle	Antifibrotic effect and inflammation response	15 weeks
Fortea et al. [22]	Unclear	Sprague-Dawley rats	Unclear	CCl_4_, cirrhosis	Saline, CCl_4_ + Saline, and CCl_4_ + enoxaparin	Survival, liver function tests, antifibrotic effect, and inflammation response	12 weeks
Assy et al. [28]	Unclear	Sprague-Dawley rats	28	TAA, hepatic cirrhosis	Controls, aspirin, and enoxaparin	Survival, liver function tests, and antifibrotic effect fibrosis	5 weeks
Li et al. [29]	Randomized controlled study	Sprague-Dawley rats	45	TAA, hepatic fibrosis	TAA, TAA + low-dose aspirin, TAA + high-dose aspirin, and TAA + enoxaparin	Liver function tests and antifibrotic effect	4 weeks
Yan et al. [30]	Randomized controlled study	C57BL/6	Unclear	CCl_4_, cirrhosis	Different groups of the enzymatically depolymerized heparins and saline	Liver function tests, antifibrotic effect, and inflammation response	8 weeks
Fujita et al. [31]	Randomized controlled study	Fischer rats	344	A CDAA diet or an HF/HC diet, NAFLD	CDAA, CDAA + aspirin, CDAA + ticlopidine, CDAA + cilostazol, CSAA	Antifibrotic effect and inflammation response	16 weeks
Abdel-Salam et al. [32]	Randomized controlled study	Sprague-Dawley rats	48	Bile duct ligated (BDL), cholestatic liver injury	Sham, BDL, BDL + UFH, BDL + nadroparin, BDL + tinzaparin, and BDL + enoxaparin	Liver function tests	3 weeks
Abe et al. [33]	Unclear	Wistar rats	Unclear	CCl_4_, hepatic fibrosis	CCl_4_ and CCl_4_ + dalteparin	Antifibrotic effect	7 weeks
Lee et al. [34]	Unclear	Sprague-Dawley rats	24	TAA, hepatic fibrosis	Saline, and dabigatran etexilate	Liver function tests, antifibrotic effect, fibrin deposition, intrahepatic angiogenesis, and portal hypertension	12 weeks
Mahmoud et al. [35]	Randomized controlled study	Albino rats	24	CCl_4_, hepatic fibrosis	Control group, CCl_4_, and CCl_4_ + rivaroxaban	Liver function tests, antifibrotic effect, and inflammation response	6 weeks
Mahmoud et al. [36]	Randomized controlled study	Albino rats	56	CCl_4_, hepatic fibrosis	Normal control, fibrosis control, dabigatran-treated, and clopidogrel-treated group	Liver function tests, antifibrotic effect, and inflammation response	6 weeks
Liu et al. [37]	Unclear	Sprague-Dawley rats	27	CCl_4_, hepatic fibrosis	Control group, CCl_4_, and CCl_4_ + aspirin	Liver function tests, antifibrotic effect, and inflammation response	6 weeks

ATIII, antithrombin III; BDL, bile duct ligated; CDAA, choline-deficient, L-amino acid-defined; CCl_4_, carbon tetrachloride; CSAA, choline-sufficient l-amino acid; HF/HC, high-fat high-calorie; L-amino acid-defined; DMN, dimethylnitrosamine; LAAH, low anticoagulant activity heparin; LH, low molecular weight heparin; LHP, low molecular weight heparinepluronic nanogel; NAFLD, nonalcoholic fatty liver disease; TAA, thioacetamide; UFH, unfractionated heparin.

**Table 2:** The grading of the quality of evidence for each outcome.

Certainty assessment							No. of patients		Effect		Certainty
No. of studies	Study design	Risk of bias	Inconsistency	Indirectness	Imprecision	Other considerations	Anticoagulant therapies	Control	Relative (95% CI)	Absolute (95% CI)	
Survival											
4	Randomized trials	Serious^a^	Not serious	Not serious	Not serious	None	143/197 (72.6%)	141/193 (73.1%)	RR 1.03 (0.94 to 1.13)	22 more per 1,000 (from 44 fewer to 95 more)	⊕⊕⊕⃝ moderate
Fibrosis evaluations by METAVIR fibrosis score system											
3	Randomized trials	Very serious^a^	Serious^b^	Not serious	Not serious	Publication bias strongly suspected^c^	37/61 (60.7%)	59/59 (100.0%)	RR 0.66 (0.47 to 0.94)	340 fewer per 1,000 (from 60 fewer to 530 fewer)	⊕⃝⃝⃝ very low
Collagen deposition											
4	Randomized trials	Very serious^d^	Very serious^e^	Not serious	Not serious	Publication bias strongly suspected^c^	142	120	—	MD −4.1 (−12.42, 4.23)	⊕⃝⃝⃝ very low
Portal pressure											
3	Randomized trials	Very serious^a^	Not serious	Not serious	Not serious	None	93	87	—	MD −1.39 (−2.33, −0.44)	⊕⊕⃝⃝ low
ALT											
10	Randomized trials	Serious^f^	Very serious^g^	Not serious	Not serious	Publication bias strongly suspected^c^	217	196	—	MD −82.7 (−107.36, −58.04)	⊕⃝⃝⃝ very low
AST											
7	Randomized trials	Serious^d^	Very serious^h^	Not serious	Not serious	Publication bias strongly suspected^c^	141	130	—	MD −186.12 (−254.90, −117.33)	⊕⃝⃝⃝ very low
Total bilirubin											
8	Randomized trials	Very serious^d^	Very serious^h^	Not serious	Not serious	None	146	134	—	MD −0.96 (−1.46, −0.46)	⊕⃝⃝⃝ very low
Albumin											
6	Randomized trials	Very serious^a^	Very serious^i^	Not serious	Not serious	None	98	88	—	MD 0.59 (0.16, 1.10)	⊕⃝⃝⃝ very low
TNF-*α*											
1	Randomized trials	Very serious^a^	Very serious^j^	Not serious	Not serious	None	24	24	—	MD −169.69 (−257.64, −81.74)	⊕⃝⃝⃝ very low

CI, confidence interval; RR, risk ratio; MD, Mean difference; ALT, alanine aminotransferase; AST, aspartate aminotransferase. *Explanations*. ^a^ (1) All articles did not state detailed randomization method and blinding. (2) Due to poor description about experiment design and methods, many items (concealed allocation and selected outcome assessment, etc.) were “unclear.” ^b^*I*^2^ = 71%. ^c^Sample size of the included studies was small and the funnel plot was asymmetric. ^d^ (1) All articles did not state a detailed randomization method. (2) Only one study stated blinding. (3) Due to poor description about experiment design and methods, many items (concealed allocation and selected outcome assessment, etc.) were “unclear.” ^e^*I*^2^ = 98%. ^f^ (1) 40% articles did not state randomization and all articles did not state a detailed method. (2) Only one study stated blinding. (3) Due to poor description about experiment design and methods, many items (concealed allocation and selected outcome assessment, etc.) were “unclear.” ^g^*I*^2^ = 97%. ^h^*I*^2^ = 98%. ^i^*I*^2^ = 94%. ^j^*I*^2^ = 80%.

**Table 3:** Subgroup analyses for fibrosis evaluation indicated by the METAVIR fibrosis score system.

Group/subgroup	Weight (%)	Effect size		Heterogeneity for each subgroup	
		RR	95% CI	*I * ^2^ (%)	*p*
All experiments	100	0.66	(0.47, 0.94)	71	0.001
Animal model					
Liver fibrosis/cirrhosis	71.9	0.73	(0.5, 1.07)	74	0.004
Nonalcoholic fatty liver disease	28.1	0.55	(0.33, 0.93)	19	0.29
Animal species					
Male	72.1	0.65	(0.4, 1.04)	79	0.0002
Unclear	27.9	0.63	(0.44, 0.91)	0	0.79
Treatment duration (weeks)					
≤8	71.9	0.73	(0.5, 1.07)	74	0.004
>8	28.1	0.55	(0.33, 0.93)	19	0.29
Treatment timing					
Simultaneous injection at model induction	56.0	0.61	(0.46, 0.81)	0	0.65
Injection after model induction	44.0	0.79	(0.46, 1.35)	81	0.005
Anticoagulation type					
Low molecular weight heparin (enoxaparin)	34.5	0.83	(0.5, 1.4)	76	0.04
Antiplatelet agents (aspirin, ticlopidine and cilostazol)	65.5	0.58	(0.37, 0.91)	61	0.03

CI, confidence interval; RR, risk ratio.

**Table 4:** Subgroup analyses for portal pressure.

Group/subgroup	Weight (%)	Effect size		Heterogeneity for each subgroup	
		MD	95% CI	*I* ^2^ (%)	*p*
All experiments	100	−1.39	(−2.33, −0.44)	45	0.07
Animal model					
Liver cirrhosis	100	−1.39	(−2.33, −0.44)	45	—
Other	0	—	—	—	—
Animal species					
Male	94.6	−1.25	(−2.27, −0.23)	56	0.03
Unclear	5.4	−3.28	(−7.11, 0.56)	0	0.66
Treatment duration (weeks)					
≤8	100	−1.39	(−2.33, −0.44)	45	—
>8	0	—	—	—	—
Treatment timing					
Simultaneous injection at model induction	21.8	0.67	(−4.92, 6.26)	90	0.001
Injection after model induction	78.2	−1.75	(−2.46, −1.03)	0	0.81
Anticoagulation type					
Low molecular weight heparin (enoxaparin)	94.6	−1.25	(−2.27, −0.23)	56	0.03
Novel oral anticoagulants (rivaroxaban)	5.4	−3.28	(−7.11, 0.56)	0	0.66

CI, confidence interval; MD, mean difference.

**Table 5:** Analyses for the effect of different anticoagulant therapies on the indices of hepatic damage.

Anticoagulant therapies	Effect estimates of indices of hepatic damage							
	ALT		AST		Albumin		Total bilirubin	
Antiplatelet agents	MD −242.29, 95% CI (−409.06, −75.51)	*p*=0.004	MD −1195.30, 95% CI (−1356.24, −1034.36)	*p* < 0.001	MD 1.10, 95% CI (0.86, 1.34)	*p* < 0.001	MD −1.79, 95% CI (−2.00, −1.59)	*p* < 0.001

LMWHs	MD −10.41, 95% CI (−24.45, 3.63)	*p*=0.15	MD −25.17, 95% CI (−46.52, −3.83)	*p*=0.02	MD −0.01, 95% CI (−0.19, 0.17)	*p*=0.91	MD −1.16, 95% CI (−2.18, −0.13)	*p*=0.03

Factor Xa inhibitor (rivaroxaban)	MD −133.66, 95% CI (−412.73, 145.40)	*p*=0.35	MD −329.49, 95% CI (−1053.49, 394.52)	*p*=0.37	MD 0.90, 95% CI (0.66, 1.13)	*p* < 0.001	MD −0.57, 95% CI (−1.69, 0.54)	*p*=0.31

Standard heparin	MD −47.46, 95% CI (−107.27, 12.34)	*p*=0.12	MD 23.42, 95% CI (−26.43, 73.27)	*p*=0.36	MD 1.77, 95% CI (0.10, 3.44)	*p*=0.04	MD 1.34, 95% CI (0.01, 2.67)	*p*=0.05

Thrombin inhibitor	MD −300.97, 95% CI (−864.44, 262.71)	*p*=0.30	MD −486.86, 95% CI (−1505.52, 531.81)	*p*=0.35	MD 1.00, 95% CI (0.77, 1.23)	*p* < 0.001	MD −0.90, 95% CI (−2.56, 0.77)	*p*=0.29

CI, confidence interval; MD, mean difference; LMWHs, low molecular weight heparin; ALT, alanine aminotransferase; AST, aspartate aminotransferase.

## Data Availability

The datasets used in the study are available from the corresponding author upon reasonable request.

## Abbreviations

AC: Anticoagulant
ALT: Alanine aminotransferase
AST: Aspartate aminotransferase
BDL: Bile duct ligation
CCl_4_: Carbon tetrachloride
CDAA: Choline-deficient L-amino acid-defined
DMN: Dimethylnitrosamine
ERK: Extracellular signal-regulated kinase
HSC: Hepatic stellate cell
HF/HC: High-fat high-calorie
MMP-2: Matrix metalloproteinase-2
MD: Mean difference
NAFLD: Nonalcoholic fatty liver disease
NO: Nitric oxide
NOAC: Novel direct oral anticoagulant
PVT: Portal vein thrombosis
PP: Portal pressure
RR: Risk ratio
SD: Standard deviation
SEM: Standard error of the mean
TAA: Thioacetamide
TGF-*β*: Transforming growth factor-beta
TIMP-1: Tissue inhibitor of metalloproteinase-1
TNF-*α*: Tumor necrosis factor-alpha.

## Supplementary Materials

Supplementary Materials Table S1: completed PRISMA checklist reporting. Table S2: detailed characteristics of selected publications. Table S3: risk of bias assessment for all studies.
