# Supplementary material for: Effects of Anticoagulants on Experimental Models of Established Chronic Liver Diseases: A Systematic Review and Meta-Analysis
Source: Can J Gastroenterol Hepatol. 2020 Dec 11;2020:8887574. doi: 10.1155/2020/8887574 (PMC7749775; doi:10.1155/2020/8887574)
Supplement: Supplementary Materials — Table S1: completed PRISMA checklist reporting. Table S2: detailed characteristics of selected publications. Table S3: risk of bias assessment for all studies. [file 8887574.f1.zip › 8887574.f1/Table S2 (2).docx]

| TABLE S2: The detailed characteristics of all included publications. | | | | | | | | | | | | | | | | | | | | |
| --- | --- | --- | --- | --- | --- | --- | --- | --- | --- | --- | --- | --- | --- | --- | --- | --- | --- | --- | --- | --- |
| Author (year) | Journal | Country/region | Study design | Animal species/strains | Animal gender | Weight of animal | Number of animal | Animal model | Method for animal model | Route of anticogulation administration | Anticogulant type | Treatment duration (weeks) | Anti-fibrotic effects | Liver function preservation | Intrahepatic microthrombosis reduction | Portal pressure reduction | Effect on HSC proliferation *in vitro* | Effect on liver sinusoidal endothelial dysfunction | Effect of inflammatory responses | Data could be extracted |
| Kassel KM 2012 [12] | The American Journal of Pathology | Kansas | Unclear | C57BL/6 | Male | Six-week-old | Unclear | NAFLD | A western diet | Feed | Argatroban | Follow-injection, 4 | Yes | NA | NA | NA | NA | NA | Yes | No |
| Li W 2006 [13] | Hepatology Research | China | Unclear | Sprague-Dawley rats | Male | 300±30g | 52 | Hepatic fibrosis | CCL4 and porcine serum | Subcutaneous injection of CCL4 and peritoneal cavity injection of porcine serum | LAAH | Co-injection, 10 | Yes | Yes | NA | NA | Yes | NA | NA | Yes |
| Lee JH 2011 [16] | Biomaterials | South Korea | Unclear | Sprague-Dawley rats | Unclear | 200g | 24 | Hepatic fibrosis | 1% DMN | Intraperitoneal injection | LH and LHP | Co-injection, 4 | Yes | Yes | NA | NA | NA | NA | NA | Yes |
| Vilaseca M 2017 [17] | Hepatology | Spain | Randomized controlled study | Wistar rats/Sprague-Dawley rats | Unclear | 50-75g (Wistar rats)/150-200g (Sprague-Dawley rats) | Unclear | Cirrhosis | CCl4 and TAA | Inhalation of CCL4 and intraperitoneal injection of TAA | Rivaroxaban | Follow-gavage, 2 | Yes | No | Yes | yes | No | Yes | NA | Yes |
| Cerini F 2015 [21] | Journal of Hepatology | Spain | Randomized controlled study | Wistar rats | Male | 50-75g (Wistar rats)/ 150-200g (Sprague-Dawley rats) | Unclear | Cirrhosis | CCl4 and TAA | Inhalation of CCL4 and intraperitoneal injection of TAA | Enoxaparin | Follow-injection, 24hours/1 week/3 weeks | Yes | No | Yes | Yes | Yes | NA | No | Yes |
| Fortea JI 2018 [22] | Liver international | Spain | Unclear | Sprague-Dawley rats | Male | 130-200g/250g/75-100g | 171 | Cirrhosis | CCl4 and BDL | Oral gavage and inhalation of CCl4, and BDL | Enoxaparin | Co-injection, unclear | No | No | NA | No | NA | NA | No | Yes |
| Assy N 2007 [28] | Digestive Diseases and Sciences | Israel | Unclear | Sprague-Dawley rats | Unclear | 200-250g | 33 | Cirrhosis | TAA | Oral gavage and intraperitoneal injection | Aspirin and enoxaparin | Co-injection, 5 | Yes | Yes | NA | NA | NA | NA | NA | Yes |
| Li CJ 2017 [29] | World Journal of Gastroenterology | China | Randomized controlled study | Sprague-Dawley rats | Male | 200-250g | 45 | Hepatic fibrosis | TAA | Intraperitoneal injection | Aspirin and enoxaparin | Follow-injection, 4 | Yes | No | NA | NA | NA | NA | NA | Yes |
| Yan Y 2017 [30] | Frontiers in Pharmacology | China | Randomized controlled study | C57BL/6 mice | Male | 20-25g | Unclear | Hepatic fibrosis | CCl4 | Intraperitoneal injection | The enzymatically depolymerized heparins | Co-injection, 8 | Yes | Yes | NA | NA | NA | NA | Yes | No |
| Fujita K 2008 [31] | Hepatology | Japan | Randomized controlled study | Fischer rats | Male | 150-160g | 344 | NAFLD | CDAA or HF/HC diet | Oral gavage | Aspirin, ticlopidine and cilostazol | Co-injection, 16 | Yes | Yes | NA | NA | NA | NA | Yes | Yes |
| Abdel-Salam OM 2005 [32] | Pharmacological Research | Egypt | Randomized controlled study | Sprague-Dawley rats | Male | 130-150g | 48 | Cholestatic liver injury | BDL | Subcutaneous injection | Heparin sodium, nadroparin, tinzaparin, and enoxaparin | Follow-injection, 3 | Yes | Yes | NA | NA | NA | NA | Yes | Yes |
| Abe W 2007 [33] | Journal of Hepatology | Japan | Unclear | Wistar rats | Female | 200g | Unclear | Hepatic fibrosis | CCL4 | Intraperitoneal injection | Dalteparin | Co-injection, 7 | Yes | No | NA | NA | Yes | NA | No | No |
| Lee KC et al. 2019 [34] | Digestive Diseases and Sciences | Taiwan | Unclear | Sprague-Dawley rats | Male | 200–250g | 24 | Hepatic fibrosis | TAA | Oral gavage | Dabigatran etexilate | Co-injection, 4 | Yes | No | NA | Yes | Yes | NA | NA | Yes |
| Mahmoud NI et al. 2019 [35] | Journal of Biochemical and Molecular Toxicology | Egypt | Randomized  Controlled study | Albino rats | Male | 230 ± 20g | 24 | Hepatic fibrosis | CCl4 | Oral administration | Rivaroxaban | Co-injection, 6 | Yes | Yes | NA | NA | NA | NA | Yes | Yes |
| Mahmoud NI et al. 2019 [36] | Life Sciences | Egypt | Randomized  Controlled study | Albino rats | Male | 200–250g | 56 | Hepatic fibrosis | CCl4 | Oral administration | Dabigatran and clopidogrel | Co-injection, 6 | Yes | Yes | NA | NA | NA | NA | NA | Yes |
| Liu Y et al. 2020 [37] | Aging | China | Unclear | Sprague-Dawley rats | Male | 180 ± 15g | 27 | Hepatic fibrosis | CCl4 | Unclear | Aspirin | Co-injection, 6 | Yes | Yes | NA | NA | Yes | NA | Yes | No |
| BDL, Bile duct ligated; CCl4, Carbon tetrachloride; CDAA, Choline-deficient, L-amino acid-defined; HF/HC, High-fat high-calorie; LAAH, Low anticoagulant activity heparin; LH, Low molecular weight heparin; LHP, Low molecular weight heparinepluronic nanogel; NAFLD, Nonalcoholic fatty liver disease; NA, not appliable; TAA, Thioacetamide. | | | | | | | | | | | | | | | | | | | | |
