# Supplementary material for: Effects of Anticoagulants on Experimental Models of Established Chronic Liver Diseases: A Systematic Review and Meta-Analysis
Source: Can J Gastroenterol Hepatol. 2020 Dec 11;2020:8887574. doi: 10.1155/2020/8887574 (PMC7749775; doi:10.1155/2020/8887574)
Supplement: Supplementary Materials — Table S1: completed PRISMA checklist reporting. Table S2: detailed characteristics of selected publications. Table S3: risk of bias assessment for all studies. [file 8887574.f1.zip › 8887574.f1/Table S3 (2).docx]

| TABLE S3: The assessment of risk of bias for all studies. | | | | | | | | | | | | | |
| --- | --- | --- | --- | --- | --- | --- | --- | --- | --- | --- | --- | --- | --- |
| Study | Item (Yes/No/Unclear) | | | | | | | | | | | | |
|  | 1) Was the allocation sequence adequately generated and applied? | 2) Were the groups similar at baseline or were they adjusted for confounders in the analysis? | 3) Was the allocation to the different groups adequately concealed during? | 4) Were the animals randomly housed during the experiment? | 5) Were the caregivers and/or investigators blinded from knowledge which intervention each animal received during the experiment? | 6) Were animals selected at random for outcome assessment? | 7) Was the outcome assessor blinded? | 8) Were incomplete outcome data adequately addressed? | 9) Are reports of the study free of selective outcome reporting? | 10) Was the study stated randomization at outcome assessment? | 11) Was the study stated randomization at allocation level? | 12) Was the study state the source of funding? | 13) Was there a conflict of interest statement? |
| Kassel KM et al 2012 [12] | Unclear | Yes | Unclear | Unclear | Unclear | Unclear | Unclear | Unclear | Unclear | Unclear | No | Yes | Unclear |
| Li W et al.2006 [13] | Unclear | Yes | Unclear | Yes | Unclear | Unclear | Unclear | Unclear | Unclear | Unclear | No | Yes | Unclear |
| Lee JH et al.2011 [16] | Unclear | Unclear | Unclear | Yes | Unclear | Unclear | Unclear | Unclear | Unclear | Yes | No | Yes | Unclear |
| Vilaseca M et al. 2017 [17] | Unclear | Unclear | Unclear | Yes | Unclear | Unclear | Unclear | Unclear | Unclear | Unclear | Yes | Yes | Yes |
| Cerini F et al. 2015[21] | Unclear | Yes | Unclear | Yes | Unclear | Unclear | Unclear | Yes | Unclear | Unclear | Yes | Yes | No |
| Fortea JI et al. 2018 [22] | Unclear | Yes | Unclear | Yes | Unclear | Unclear | Unclear | Unclear | Unclear | Unclear | No | Yes | No |
| Assy N et al.2007 [28] | Unclear | Unclear | Unclear | Yes | Unclear | Unclear | Unclear | Yes | Unclear | Unclear | No | No | Unclear |
| Li CJ et al. 2017 [29] | Unclear | Yes | Unclear | Yes | Unclear | Yes | Unclear | Unclear | Unclear | Yes | Yes | No | Unclear |
| Yan Y et al. 2017 [30] | Unclear | Yes | Unclear | Unclear | Unclear | Unclear | Unclear | Unclear | Unclear | Unclear | Yes | Yes | No |
| Fujita K et al. 2008 [31] | Unclear | Yes | Unclear | Yes | Unclear | Unclear | Unclear | No | Unclear | Unclear | Yes | Yes | No |
| Abdel-Salam OM et al. 2005 [32] | Unclear | Yes | Unclear | Yes | Yes | Unclear | Unclear | Unclear | Unclear | Unclear | Yes | No | Unclear |
| Abe W et al. 2007 [33] | Unclear | Yes | Unclear | Unclear | Unclear | Unclear | Unclear | Unclear | Unclear | Unclear | No | Yes | Unclear |
| Lee KC et al. 2019 [34] | Unclear | Yes | Unclear | Unclear | Unclear | Unclear | Unclear | Unclear | Unclear | Yes | No | Yes | No |
| Mahmoud NI et al. 2019 [35] | Unclear | Yes | Unclear | Unclear | Unclear | Unclear | Unclear | Unclear | Unclear | Unclear | Yes | No | No |
| Mahmoud NI et al. 2019 [36] | Unclear | Yes | Unclear | Unclear | Unclear | Unclear | Unclear | Unclear | Unclear | Unclear | Yes | No | No |
| Liu Yet al. 2020 [37] | Unclear | Yes | Unclear | Unclear | Unclear | Unclear | Unclear | Unclear | Unclear | Unclear | No | Yes | No |
